# Inhibition of SALL4 reduces tumorigenicity involving epithelial-mesenchymal transition via Wnt/β-catenin pathway in esophageal squamous cell carcinoma

**DOI:** 10.1186/s13046-016-0378-z

**Published:** 2016-06-21

**Authors:** Jing He, Mingxia Zhou, Xinfeng Chen, Dongli Yue, Li Yang, Guohui Qin, Zhen Zhang, Qun Gao, Dan Wang, Chaoqi Zhang, Lan Huang, Liping Wang, Bin Zhang, Jane Yu, Yi Zhang

**Affiliations:** Biotherapy Center, the First Affiliated Hospital of Zhengzhou University, Zhengzhou, Henan 450052 China; Department of Oncology, the First Affiliated Hospital of Zhengzhou University, Zhengzhou, Henan 450052 China; Department of Hematology/Oncology, School of Medicine, Northwestern University, Chicago, IL60611 USA; Department of Internal Medicine, College of Medicine, University of Cincinnati, 231 Albert Sabin Way, Cincinnati, OH 45267 USA; School of Life Sciences, Zhengzhou University, Zhengzhou, Henan 450001 China; Henan Key Laboratory for Tumor Immunology and Biotherapy, Zhengzhou, Henan 450052 China

**Keywords:** SALL4, Esophageal squamous cell carcinoma (ESCC), Stemness, Epithelial-mesenchymal transition (EMT), Prognostic marker

## Abstract

**Background:**

Growing evidence suggests that SALL4 plays a vital role in tumor progression and metastasis. However, the molecular mechanism of SALL4 promoting esophageal squamous cell carcinoma (ESCC) remains to be elucidated.

**Methods:**

The gene and protein expression profiles- were examined by using quantitative real-time PCR, immunohistochemistry and western blotting. Small hairpin RNA was used to evaluate the role of SALL4 both in cell lines and in animal models. Cell proliferation, apoptosis and invasion were assessed by CCK8, flow cytometry and transwell-matrigel assays. Sphere formation assay was used for cancer stem cell derivation and characterization.

**Results:**

Our study showed that the transcription factor SALL4 was overexpressed in a majority of human ESCC tissues and closely correlated with a poor outcome. We established the lentiviral system using short hairpin RNA to knockdown SALL4 in TE7 and EC109 cells. Silencing of SALL4 inhibited the cell proliferation, induced apoptosis and the G1 phase arrest in cell cycle, decreased the ability of migration/invasion, clonogenicity and stemness in vitro. Besides, down-regulation of SALL4 enhanced the ESCC cells’ sensitivity to cisplatin. Xenograft tumor models showed that silencing of SALL4 decreased the ability to form tumors in vivo. Furthermore, our study demonstrated that SALL4 played a vital role in modulating the stemness of ESCC cells via Wnt/β-catenin signaling pathway and in epithelial-mesenchymal transition.

**Conclusions:**

Our results revealed that SALL4 might serve as a functional marker for ESCC cancer stem cell, a crucial marker for prognosis and an attractive candidate for target therapy of ESCC.

## Background

Esophageal cancer is the sixth leading cause of cancer-related death in the world and third in China [1, 2]. Esophageal squamous cell carcinoma (ESCC) is the predominant subtype of esophagus cancer. Almost 90 % of cases are squamous cell carcinomas in the highest risk area of Asia, especially in Henan province [3, 4]. Due to the lack of early detection, most of the patients are often diagnosed with advanced disease, causing the prognosis remains dismal and large probability of recurrent disease. Recent data indicated that 5-year overall survival was only 17 % in ESCC [5]. Thus, there is an urgent need to explore the underlying molecular mechanisms and the new potential treatment targets. Malignancies arise from a small population of stem-cell-like cancer cells. These cells are known as cancer stem cells (CSCs). CSCs were widely studied in various tumors and CSCs targeted therapy could be a great help for ESCC treatment [6]. CSCs have high tumor-initiating, self-renewal and differentiation ability. Moreover, CSCs are resistant to chemotherapy and radiotherapy and responsible for cancer recurrence and distant metastasis [7, 8]. Markers that identify CSCs have been found in some tumors [9, 10]. However, the candidate biomarkers in human ESCC are limited.

The sal-like protein 4 (SALL4) is a member of SALL gene family. It was firstly cloned based on its sequence homology to Drosophila spalt [11]. SALL4 acts as a C2H2 zinc-finger transcription factor, plays essential roles in maintaining pluripotency and self-renewal of embryonic and hematopoietic stem cells [12–14]. It’s also engaged in maintaining stemness state and survival by forming a SALL4, Oct4, Sox2 and Nanog transcription regulation feedback loop [15, 16]. SALL4 was firstly described in leukemogenesis, with constitutive expression in human acute myeloid leukemia [17]. Subsequently, SALL4 expression has been found in various types of solid tumors, including lung cancer, endometrial cancer, gastric cancer and liver cancer [18–21].

Although emerging evidence reveals that SALL4 is involved in carcinogenesis, the functional roles of SALL4 in ESCC are still unknown. In this study, we aimed to undertake a detailed analysis of SALL4 expression in ESCC and explore its underlying mechanism. We further investigated the function of SALL4 in ESCC in vitro and in vivo, and characterized the role of SALL4 in regulating the proliferation, apoptosis, cell cycle, migration, invasion, clonogenicity and stemness of ESCC cells. Our studies also suggested depletion of SALL4 induced a mesenchymal-epithelial transition (MET) phenotype via Wnt/β-catenin signaling pathway. Taken together, our studies indicate that SALL4 plays a pivotal role during ESCC development and progression and might serve as a potential target of ESCC treatment.

## Methods

### Patients and tumor samples

One hundred and thirty-three paired ESCC tissues and their matching adjacent non-cancerous tissues (located more than 5 cm away from cancer tissues) were obtained from The First Affiliated Hospital of Zhengzhou University. All patients were not received any therapeutic intervention such as chemo- and radiotherapy. The samples used in the study were approved by the ethics committee of the First Affiliated Hospital of Zhengzhou University and all patients provided written informed consent. The clinicopathologic characteristics were evaluated including gender, age, stage, histological grade, lymph nodes metastasis and tumor invasion. The patients were staged according to the TNM staging system and all samples were confirmed by pathological analysis.

### Cell culture and ESCC cell lines

Normal esophageal epithelium cell line Het-1a, human ESCC cell lines TE1, TE7, EC1, EC109, EC9706, KYSE70 and KYSE450 were preserved in our laboratory and maintained in RMPI 1640 (Hyclone, USA) supplemented with 10 % fetal bovine serum (FBS, Hyclone, USA), 100 units/mL of penicillin, and 100 μg/ml of streptomycin at 37 °C, 5 % CO_2_ in a humidified incubator. Cells infected with lentivirus were sorted by flow cytometry. Then cells were harvested and cultured for subsequent functional studies.

### RNA extraction and quantitative real-time polymerase chain reaction (PCR)

Total RNA was extracted from ESCC cells and tissues with TRIzol reagend (Invitrogen Corporation, Carlsbad, CA) according to manufacturer’s instructions. The concentration and purity of RNA were detected using NanoDrop 2000 (Thermo Scientific). The First-strand cDNA was synthesized from 1 μg of total RNA using PrimeScript RT reagent Kit With gDNA Eraser (TaKaRa, Japan). Quantitative real-time PCR was performed using SYBR Premix Ex Taq II (TaKaRa, Japan) in Agilent Mx3005P. Each experiment was performed in triplicate. Glyceraldehyde-3-phosphate dehydrogenase (GAPDH) was used as an endogenous control for normalization.

### Immunohistochemistry

Immunohistochemistry was performed according to standard protocols. SALL4 expression was scored according to the percentage of positive stained-tumor cells and the intensity of staining for SALL4. The percentage scoring of immunoreactive tumor cells was as follows: 0 (<10 %), 1 (10–40 %), 2 (40–70 %) and 3 (>70 %). The staining intensity was visually scored and stratified as follows: 0 (negative), 1 (yellowish), 2 (light brown), 3 (dark brown). A final immunoreactivity score was obtained for each case by multiplying the percentage and the intensity score. High SALL4 group had scores equal to or exceeded the median level of expression, whereas low SALL4 group with scores less than the medium level of SALL4 expression. In addition, SALL4 expression in ESCC tissues was scored by a pathologist and two researchers independently.

### Western blotting

Total cell proteins were extracted in RIPA lysis buffer. The protein concentration was determined by using BCA method (Biyuntian, Jiangsu, China). Equal amount of proteins were loaded on a 10 % SDS-PAGE (sodium dodecyl sulfate-polyacrylamide gel electrophoresis) gel. Following electrophoresis, the proteins were blotted to a PVDF membrane. The membrane was blocked in 5 % non-fat milk and then incubated with primary antibodies overnight at 4 °C and secondary antibody reactions for 2 h at 37 °C. Sources of primary antibodies were: anti-SALL4, anti-Vimentin and anti-E-cadherin (Abcam, Cambridge, MA, USA), anti-β-catenin, anti-wnt3a, anti-β-actin antibodies (Cell Signaling Technology, USA)

### Lentiviral generation and cell sorting

Stably knockdown of SALL4 in TE7 and EC109 cells by shRNA was achieved using pGLV-H1-GFP + Puro vector plasmid purchased from GenePharma (GenePharma, Shanghai, China). For viral transductions, 500 μL of the pGLV-H1-shControl or pGLV-H1-shSALL4 lentiviruses were transfected with TE7 cells, and the expression of SALL4 was confirmed by real-time RT-PCR and western blotting. All the inserted sequences were confirmed by DNA sequencing. After 48 h, the transfected cells were sorted by flow cytometry (Beckman MoFlo XDP, Brea, CA, USA) according to the expression of green fluorescent protein (GFP).

### Cell proliferation assay

The proliferation of TE7 and EC109 cells was examined via CCK-8 (Dojindo, Japan) assay. Briefly, cells were counted at 1000 cells in 100 μL medium per well plated in 96-well plates and grown under normal conditions. Then for 5 d, every 24 h a batch of cells were stained with 10 μL of CCK-8 reagent at 37 °C for 2 h, absorbance at 450 nm was measured to calculate the number of viable cells. All assays were performed in quadruplicate. The data were expressed as the mean values ± SD of 4 wells per treatment.

### Flow cytometrical evaluation of apoptosis

After 48 h of transfection with scramble shRNA and shSALL4, TE7 and EC109 cells were harvested and washed with ice-cold PBS twice. And then cells were suspended in the Annexin V-binding buffer to a final concentration of 10^6^ cells/mL. Thereafter, cells were incubated with AlexaFluor647 AnnexinV (Biolegend, USA) for 15 min at 4 °C in the dark, and PI (Sigma, USA) was added. Samples were immediately analyzed by flow cytometry (BD FACSCantoll).

### Cell cycle analysis

Cells plated onto the 6-well plate were trypsinized without EDTA and washed with pre-cooling normal saline. Then the cells were fixed by 70 % alcohol overnight at 4 °C. Cell cycle analysis was performed using RNaseA and PI staining by flow cytometry. The cells in G0/G1, S and G2/M phases were measured using FlowJo software.

### Transwell migration and invasion assays in vitro

Migration assay was performed by a filter of transwell system (8.0 μm Pore Size, 24-well insert) and Matrigel (BD, USA) was plated to the wells and used for invasion assay. 5 × 10^4^ cells with FBS-free media were added to the upper chamber and 600 μL of medium with FBS was added to the lower chamber. Then cells were incubated under standard culture conditions for 24 h (migration assay) or 48 h (invasion assay). Cells remaining on the top of membrane were removed by using cotton swab and the migrated or invaded tumor cells were stained by 0.1 % crystal violet solution for calculating analysis.

### Colony-formation assay

Five hundred cells per well were seeded into 6-well plate and incubated at 37 °C in a 5 % CO_2_ humidified incubator for 10 d. The medium was changed at 3 d intervals. At the end of the assay, the cultures were fixed with 4 % paraformaldehyde and stained with crystal violet.

### Sphere formation assay

The sorted TE7 and EC109 cells were grown in DMEM/F12 medium (Invitrogen Life Technologies) supplemented with 4 μg/mL heparin (Sigma, St. Louis, MO, USA), 2 % B27 (Gibco, Life Technologies, Carlsbad, CA, USA), 20 ng/mL basic fibroblast growth factor (bFGF) and epidermal growth factor (EGF, both from PeproTech, Rocky Hill, NJ, USA), 100 IU/mL penicillin and 100 μg/mL streptomycin. Then they were seeded in 6-well ultra-low cluster plates (Corning Costar, Corning, NY, USA). After culturing for 14 d, the number of spheres was quantified by using microscopy (Leica, Wetzlar, Germany).

### Drug sensitivity assay

Cisplatin was purchased from Sigma. The sorted TE7 and EC109 cells were seeded at 3000 cells/well in 96-well plate and treated with chemotherapeutic reagents in quadruplicate. Cell viability was evaluated using CCK-8 assay following treatment with indicated dosages for 48 h, and the absorbance was measured at 450 nm using Multiskan Mk3 (Thermo Fisher Scientific, San Jose, CA, USA). The percentage of survival in treated cells was normalized with untreated controls. The apoptosis was analyzed by flow cytometry.

### Animal model

To generate a subcutaneous xenograft mouse model, 10 female BALB/c nude mice (Vital River Laboratory Animal Technology Co. Ltd, China) aged 5 weeks were randomly divided into two groups (five mice/group). Both groups received hypodermic injections of either scramble shRNA or shSALL4 TE-7 cells (5 × 10^6^ cells in 200 μL PBS). Mice were inspected every 3 d and tumor growth was evaluated by measuring the length and width of the tumor mass with calipers. Then the mice were sacrificed at 30 d after inoculation and solid tumors were removed and measured. The tumor volume was calculated by the formula: (length × width^2^)/2. All animal procedures were conducted in accordance with the Guide for the Care and Use of Laboratory Animals, and were approved by the Institutional Animal Care and Use committee of the First Affiliated Hospital of Zhengzhou University.

### Statistical analysis

Data were expressed as mean ± SD and analyzed using the Student’s t-test or chi-squared test. Paired t-test was used for paired samples. Overall survival curves were plotted according to the Kaplan–Meier method. Statistical analyses were performed using GraphPad Prism 5 software (GraphPad Software, La Jolla, CA, USA). *P* < 0.05 was considered to indicate a statistically significant difference.

## Results

### The level of SALL4 expression is up-regulated in ESCC tissues

**Fig. 1:**
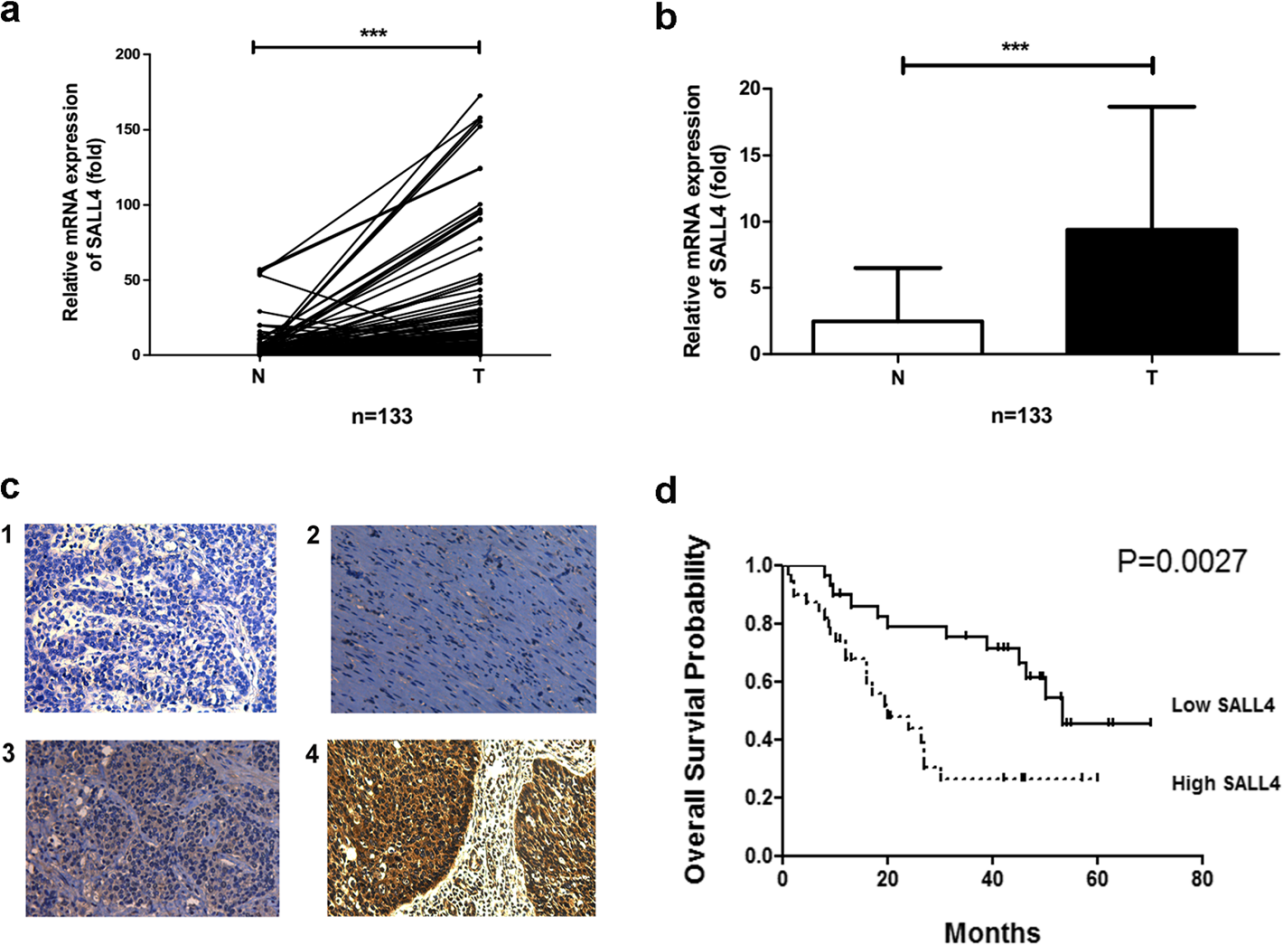
Expression of SALL4 in ESCC tissues. **a** and **b** Real-time PCR analysis of SALL4 mRNA expression in ESCC (T) and paired non-cancerous (N) tissues (***P < 0.001). **c** Representative photomicrographs of immunohistochemical staining for SALL4 in ESCC tissues and adjacent normal tissues. 1-Negative staining, 2-Normal tissues, 3-Weak staining, 4-Strong staining. **d** The Kaplan-Meier curves for the overall survival in ESCC patients with SALL4-low and SALL4-high expression (*P* = 0.0027). Original magnification: 400×

To investigate the expression of SALL4 in ESCC, one hundred and thirty-three paired ESCC tissues and adjacent non-cancerous tissues were analyzed by real-time PCR. The results showed that SALL4 expression in ESCC tissues was significantly higher than that in the matched non-cancerous tissues (*P* < 0.0001, Fig. 1a and b). Then, the correlation between SALL4 expression and patients’ clinicopathological characteristics was evaluated (Table 1). ESCC tissues with exceeded or equal to twofold of SALL4 mRNA expression (compared to adjacent normal control) were defined as the high group, while those with less than twofold of SALL4 mRNA expression were interpreted as the low group. Among the 133 ESCC cases, 102 (76.7 %) showed high level of SALL4 expression and 31 (23.3 %) showed low level of SALL4 expression in cancer tissues. Moreover, SALL4 expression was closely correlated with the stage of tumor (*P* = 0.0477) and lymph node metastasis (*P* = 0.0068). Particularly, 35 (92.1 %) of 38 ESCC patients with lymph node metastasis showed high level of SALL4 expression. Our results reveal that SALL4 expression was strongly correlated with lymph node metastasis and advanced clinical stage.

**Table 1:** Association between clinicopathological factors and SALL4 expression in ESCC patients

Parameters	Number	SALL4 expression		*P*-value
		High group	Low group	
Age				
≥60	94	75	19	0.2594
<60	39	27	12	
Gender				
Male	87	70	17	0.1965
Female	46	32	14	
Stage				
I + IIa	92	66	26	**0.0477** ^*^
IIb + III + IV	41	36	5	
Tumor invasion				
T1 + T2	64	46	18	0.2244
T3	69	56	13	
Lymph node metastasis				
N0	95	67	28	**0.0068** ^**^
N1 + N2	38	35	3	
Histological Grade				
Well differentiated	33	23	10	0.0648
Moderately differentiated	78	58	20	
Poorly differentiated	22	21	1	

* *P* <0.05, ** *P* <0.01

We further detected SALL4 protein expression in ESCC and adjoining normal tissues by immunohistochemistry. In general, the results suggested that the intensity and percentage of SALL4 immunostaining in cancer tissues were much stronger than those in adjacent non-cancerous tissues (Fig. 1c). Meanwhile, our immunohistochemistry results supported that patients with lymph node metastasis and advanced tumor stages had a stronger expression of SALL4 compared to those without lymph node metastasis and with early tumor stages. Additionally, to examine whether SALL4 expression was associated with poor prognosis, the survival analysis was performed by using Kaplan-Meier method. The 68 ESCC patients were divided into high or low group according to the SALL4 expression scoring by using immunohistochemistry. The results revealed that the overall survival probability of high group was significantly lower than those of the low group (*P* = 0.0027, Fig. 1d), the average survival time for SALL4 low expression group was 39.6 months, whereas the median survival time for SALL4 high expression group was only 18.3 months, indicating that SALL4 could serve as a potential prognostic marker for ESCC. Taken together, our results indicate that SALL4 expression is closely correlated with tumor stage, lymph node metastasis and poor survival in ESCC patients.

### SALL4 depletion decreases cell viability by inhibiting proliferation, triggering cell apoptosis and inducing cell cycle arrest in vitro

**Fig. 2:**
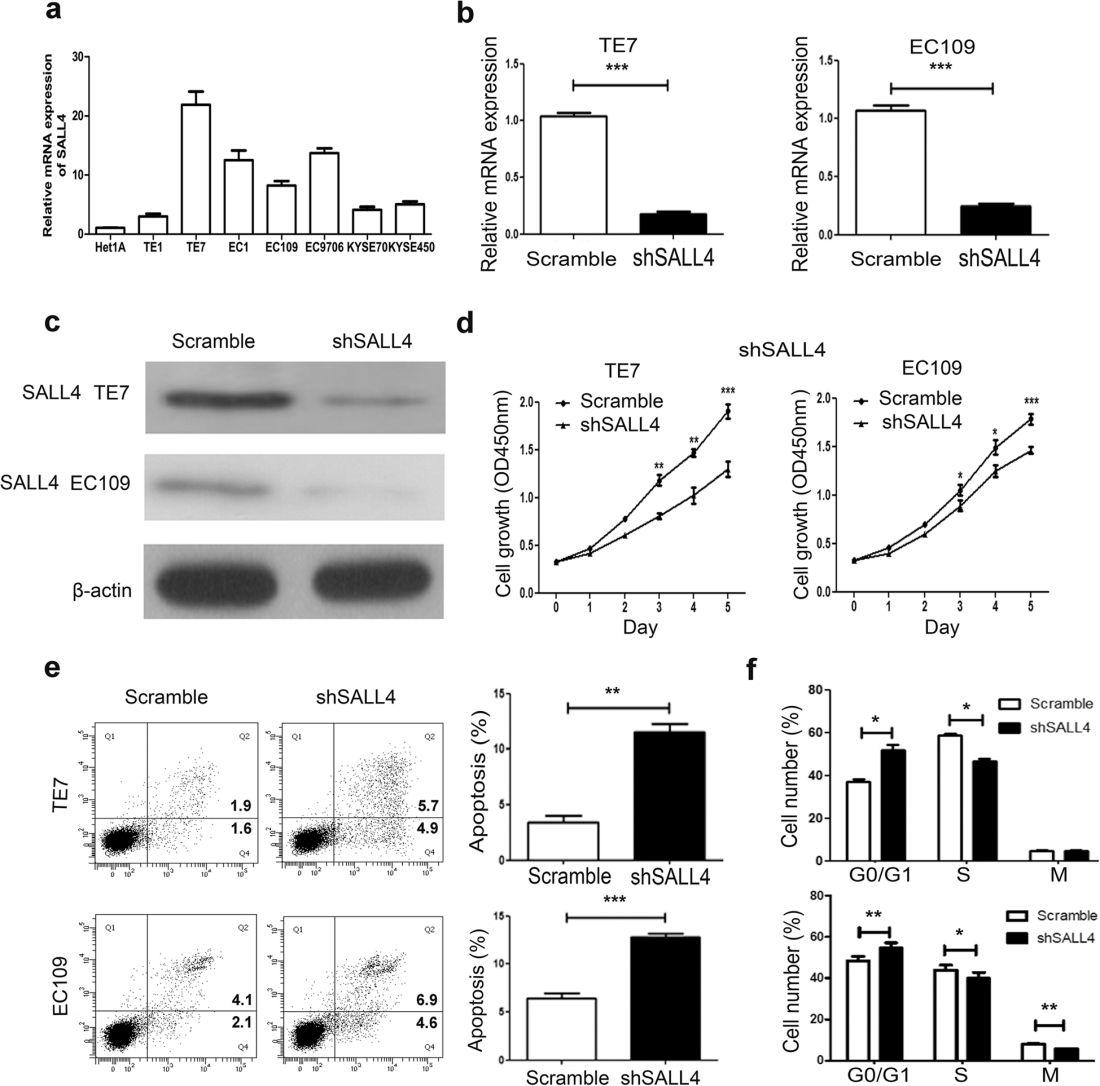
Silencing of SALL4 inhibits cell proliferation, induces apoptosis and arrests cell cycle in vitro. **a** Real-time PCR analysis of SALL4 expression in Het1A, TE1, TE7, EC1, EC109, EC9706, KYSE70, KYSE450 cell lines. **b** The mRNA level of SALL4 was verified in sorted TE7 and EC109 cells after transfection. **c** The protein level of SALL4 in sorted TE7 and EC109 cells was assessed by using Western blotting. β-actin was used as an internal control. **d** Cell viability was evaluated at indicated time points using CCK8 assay. **e** Cell apoptosis was measured by flow cytometric analysis. **f** Knock-down of SALL4 induced cell cycle arrest at G0/G1 phase. (*P < 0.05, **P < 0.01, ***P < 0.001)

To assess the biological functional role of SALL4 in ESCC, we further explored the expression of SALL4 in an immortalized esophageal epithelial cell line (Het1A) and 7 ESCC cell lines (TE1, TE7, EC1, EC109, EC9706, KYSE70 and KYSE450) by real-time PCR (Fig. 2a). Compared with the normal epithelia cell line, all ESCC cell lines showed different levels of elevation. The highest and moderate SALL4 mRNA expression cell lines TE7 and EC109 were selected for further research.

To explore the functional role of SALL4 in ESCC cells, we used a lentiviral system to generate stably SALL4 knockdown cell lines. Two short hairpin RNAs (shRNAs) designated as scramble and shSALL4 were specially designed and constructed. After transfection for 72 h, the stably transfected TE7 and EC109 cells were sorted by flow cytometry. After cultured for 2 weeks, the purities of sorted scramble and shSALL4 cells of TE7 was 97.8 and 96.1 %, respectively, the purities of sorted EC109 cells were 95.6 and 94.2 %. Real-time PCR and western blot analysis were used to confirm the knockdown efficiency of SALL4. The level of SALL4 mRNA expression was significantly reduced in shSALL4 cells compared to that in scramble cells (Fig. 2b). In addition, the suppressed expression of SALL4 protein in both sorted TE7 and EC109 cells was confirmed by using western-blot analysis (Fig. 2c). The above results demonstrated that the expression of SALL4 could be down-regulated by shRNAs specifically and effectively.

Furthermore, we analyzed the effect of shSALL4 on cell growth and apoptosis of ESCC cells. To determine the effect of down-regulation of SALL4 on cell proliferation, we performed the CCK-8 assay. The results showed that down-regulation of SALL4 significantly inhibited TE7 and EC109 cells proliferation (Fig. 2d). The effect of SALL4 depletion on the apoptosis of ESCC cells using Annexin V staining was further assessed. The percentages of Annexin V-positive cells were much higher in shSALL4 groups than that in scrambled groups (Fig. 2e). Then we investigated how the cell cycle populations altered during the apoptosis induced by the knockdown of SALL4. Cell cycle distribution analysis demonstrated that the number of cells was increased at G0/G1 phase and was decreased at S phase in shSALL4 cells, suggesting that knockdown of SALL4 induces typical cell cycle arrest at G0/G1 phase in ESCC cells (Fig. 2f). Collectively, these data suggest that the depletion of SALL4 inhibits cell proliferation, triggers cell apoptosis and induces cell cycle arrest in vitro.

### Silencing of SALL4 suppresses migration and invasion of ESCC cells in vitro

**Fig. 3:**
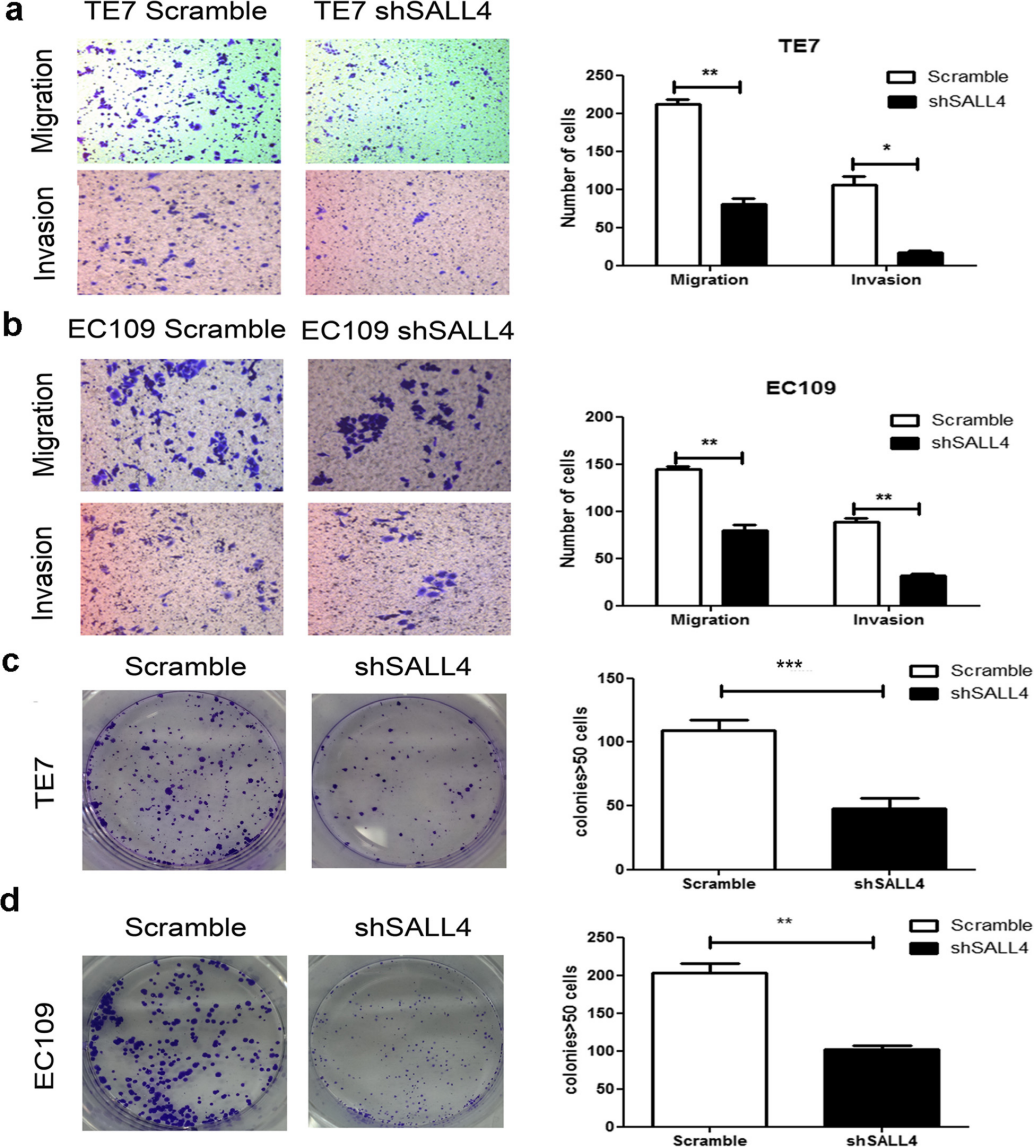
Silencing of SALL4 suppresses migration and invasion capabilities in vitro*.* **a** The migratory and invasive capabilities of TE7 and EC109 cells were evaluated by using transwell migration and invasion assay. Representative microscopic images of the bottom chamber were shown. **b** The bar graphs represented the average number of migrated cells and invaded cells on the underside of the membrane. The cell colony was decreased after transfection in both shSALL4 TE7 (**c**) and EC109 (**d**) cells. Colonies with >100 cells were quantified. Original magnification: 200×. (*P < 0.05, **P < 0.01, ***P < 0.001)

Metastasis is a central problem during cancer therapeutics, as our result has indicated that the SALL4 expression was notably associated with lymph node metastasis, thereby we aimed to asses whether loss of SALL4 could affect tumor migration and invasion ability. As shown in Fig. 3a and b, stable knock-down of SALL4 remarkably reduced the number of migrated and invaded cells to the lower side of the membrane. In addition, the migration and invasion capacities of the scramble cells were significantly greater than those of shSALL4 cells. These results indicate that SALL4 plays an essential role in the migration and invasion of ESCC.

Subsequently, the colony-formation assay was conducted. The results showed that TE7 and EC109 cells transfected with shSALL4 formed fewer colonies than that in control cells (Fig. 3c and d), suggesting that silencing SALL4 inhibits the anchorage-independent growth of ESCC cells. Taken together, these findings suggest that down-regulation of SALL4 significantly impedes the migration and invasion of ESCC cells in vitro.

### SALL4 is necessary for the maintenance of CSC properties like self-renewal and drug resistance

**Fig. 4:**
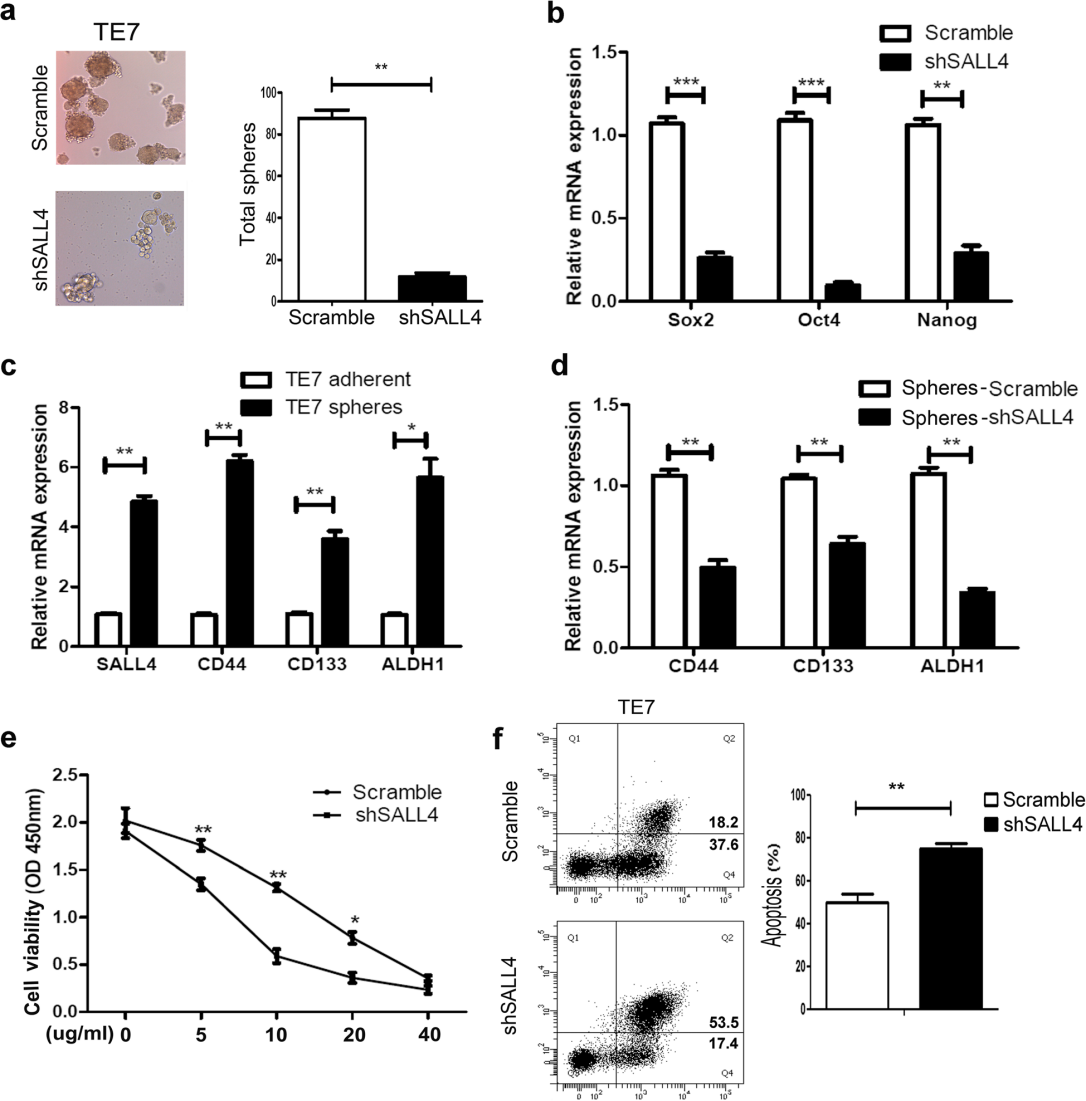
SALL4 is necessary for the maintenance of CSC properties. **a** Bright-field microscopy images of spheres generated from scramble and shSALL4 TE7 cells after the sphere-formation assay. Original magnification: 200×. **b** The expression of stemness-related markers (Sox2, Oct4, Nanog) was down-regulated in shSALL4 cells compared with scramble cells in TE7. **c** The expression of CSC markers SALL4, CD44, CD133 and ALDH1 were up-regulated in TE7 spheres than control adherent cells. **d** Real-time PCR showing CD44, CD133 and ALDH1 in spheres generated from scramble and shSALL4 TE7 cells. **e** The sensitivity of shSALL4 TE7 cells to cisplatin was increased in a concentration-dependent manner compared with scramble cells. **f** The apoptosis rate was enhanced in shSALL4 TE7 cells treated with 10 μg/mL cisplatin. (*P < 0.05, **P < 0.01, ***P < 0.001)

To further evaluate the role of SALL4 in maintenance of CSC properties, the sphere-formation assay was performed. After culturing for 14 days, the number and the size of tumor-spheres in the scramble group was obviously higher than that in the shSALL4 group (Fig. 4a), while EC109 cells showed no sphere-forming capacity at both scramble and shSALL4 groups (data not shown). In addition, the mRNA expression level of the stemness marker Sox2, Oct4, Nanog were all down-regulated in shSALL4 group, which was confirmed by using RT-PCR (Fig. 4b).

The process of sphere-forming is intended to enrich the potential CSC subpopulation, and then we characterized the expression of CSC-related markers in CSC-enriched subpopulation. The expression of SALL4 and CSC-related markers, including CD44, CD133 and ALDH1 was analyzed in spheres and adherent cells respectively. As shown in Fig. 4c, TE7 sphere-forming cells were highly expressed these CSC-related markers (CD44, CD133 and ALDH1) as well as SALL4 compared to the adherent cells. Sphere formation can be considered as a way that enriches CSC-like cells, in our study the expression of SALL4 was obviously increased in spheres. Therefore, we hypothesize SALL4 might be a potential CSC marker for ESCC. Moreover, lower expression level of CSC-related markers (CD44, CD133 and ALDH1) was found in SALL4 knockdown cells compared to that in control (Fig. 4d). These data provide proof of the role of SALL4 as a functional marker for CSCs and a regulator for the maintenance of self-renewal properties in ESCC.

Cisplatin resistance is inevitable in the process of cancer treatment and remains to be solved. Recent study showed that the existence of CSCs was responsible for chemotherapeutic failure [22]. Besides, cisplatin resistance is an impenetrable hurdle in ESCC patients. To investigate the role of SALL4 in contributing to drug resistance, we further explored whether SALL4 silencing could affect the sensitivity of TE7 cells to cisplatin treatment. Scramble and shSALL4 TE7 cells were treated with different concentrations of cisplatin, respectively. After 48 h, the CCK-8 assays showed that knockdown of SALL4 decreased cisplatin resistance and increased drug sensitivity in a dose-dependent manner (Fig. 4e). To further investigate whether cisplatin could also induce apoptosis in TE7 cells, apoptosis assay was performed using Annexin-V by Flow Cytometry analysis, and the results showed that knockdown of SALL4 could significantly enhance the drug sensitivity to cisplatin treatment and induced cell apoptosis (Fig. 4f). In summary, our data indicate that down-regulation of SALL4 increases the drug sensitivity of ESCC cells to cisplatin. These findings could be explored for future clinical application.

### Down-regulation of SALL4 promotes mesenchymal-epithelial transition (MET) via Wnt/β-catenin signaling pathway during ESCC tumorigenisis

**Fig. 5:**
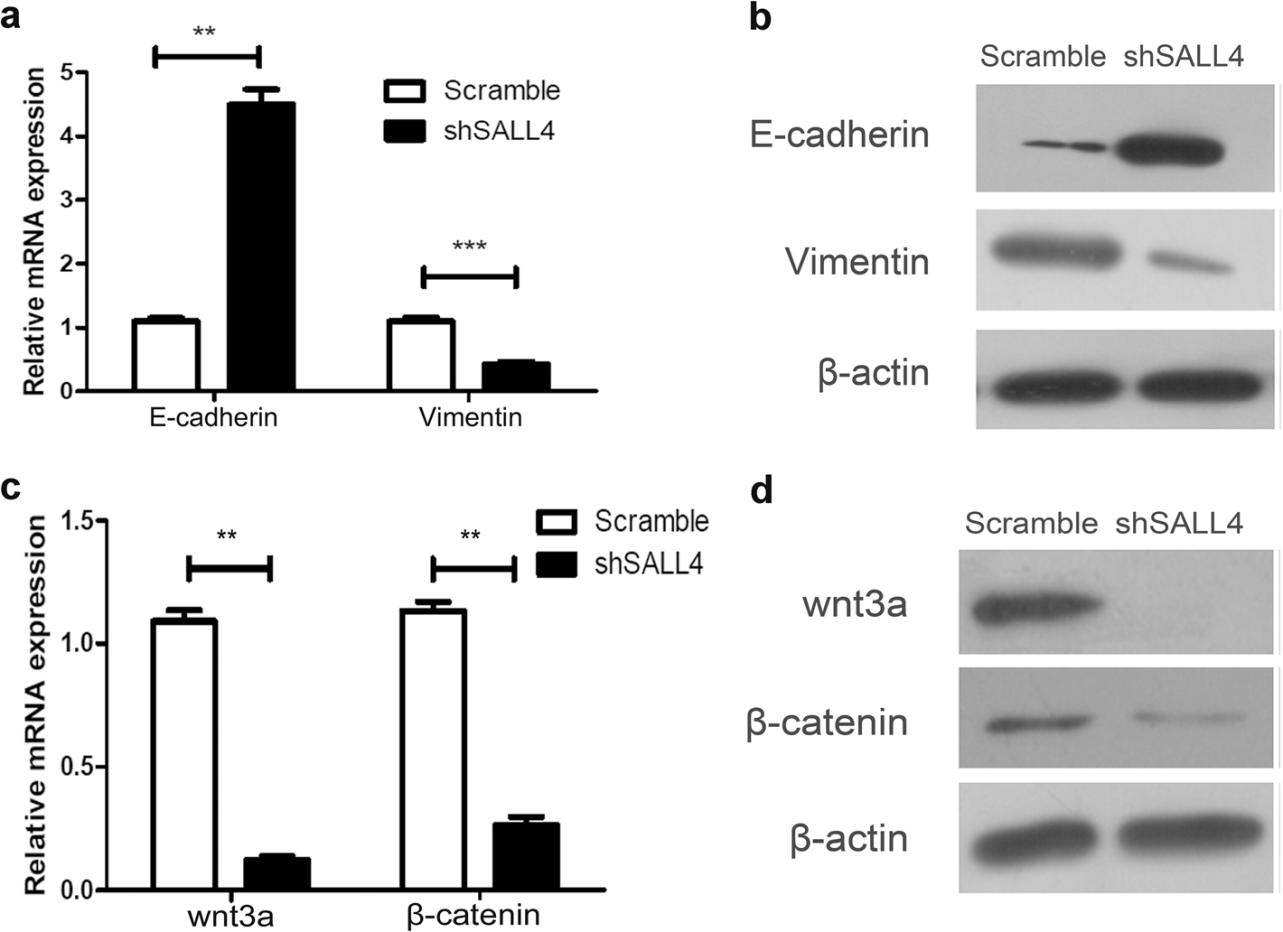
Down-regulation of SALL4 promotes mesenchymal-epithelial transition (MET) via Wnt/β-catenin signaling pathway during ESCC tumorigenisis. The expression of epithelial cell marker (E-cadherin) was elevated, while mesenchymal cell marker (Vimentin) was decreased in TE7 shSALL4 cells compared with scramble cells both in mRNA level (**a**) and protein level (**b**). The expression of the key molecules in Wnt/β-catenin pathway Wnt3a and β-catenin was down-regulated determined by real-time PCR (**c**) and western blotting (**d**) after silencing of SALL4. (**P < 0.01, ***P < 0.001)

The acquisition of epithelial-mesenchymal transition (EMT) phenotype and induction of CSC are interrelated and contributes to metastasis of cancers. Therefore, we further tested whether the down-regulation of SALL4 could have an effect on the development of EMT in ESCC cells using real-time PCR and western blotting assay. The results indicated that the mRNA and protein levels of epithelial cell marker (E-cadherin) were significantly increased. Conversely, the expression of mesenchymal cell marker (Vimentin) was decreased in shSALL4 group (Fig. 5a and b). All the data suggest that down-regulation of SALL4 promotes MET and attenuates EMT in ESCC.

The Wnt/β-catenin signaling pathway is known to play a vital role in driving the initiation of tumorigenisis and tumor growth. Activation of Wnt/β-catenin pathway can promote transcriptional changes in order to drive EMT in cancer [23]. Thus, we detected the expression of Wnt3a and β-catenin in TE7 cells with stable down-regulation of SALL4 using real-time PCR and western blotting analyses. Strikingly, our results showed that knockdown of SALL4 remarkably decreased the expression of Wnt3a and β-catenin in both mRNA (Fig. 5c) and protein level (Fig. 5d), suggesting that SALL4 could activate the Wnt/β-catenin signaling pathway to drive EMT in ESCC. In summary, our results uncover that silencing of SALL4 promotes MET via Wnt/β-catenin signaling pathway during ESCC tumorigenisis.

### Knockdown of SALL4 inhibits tumor formation in vivo

**Fig. 6:**
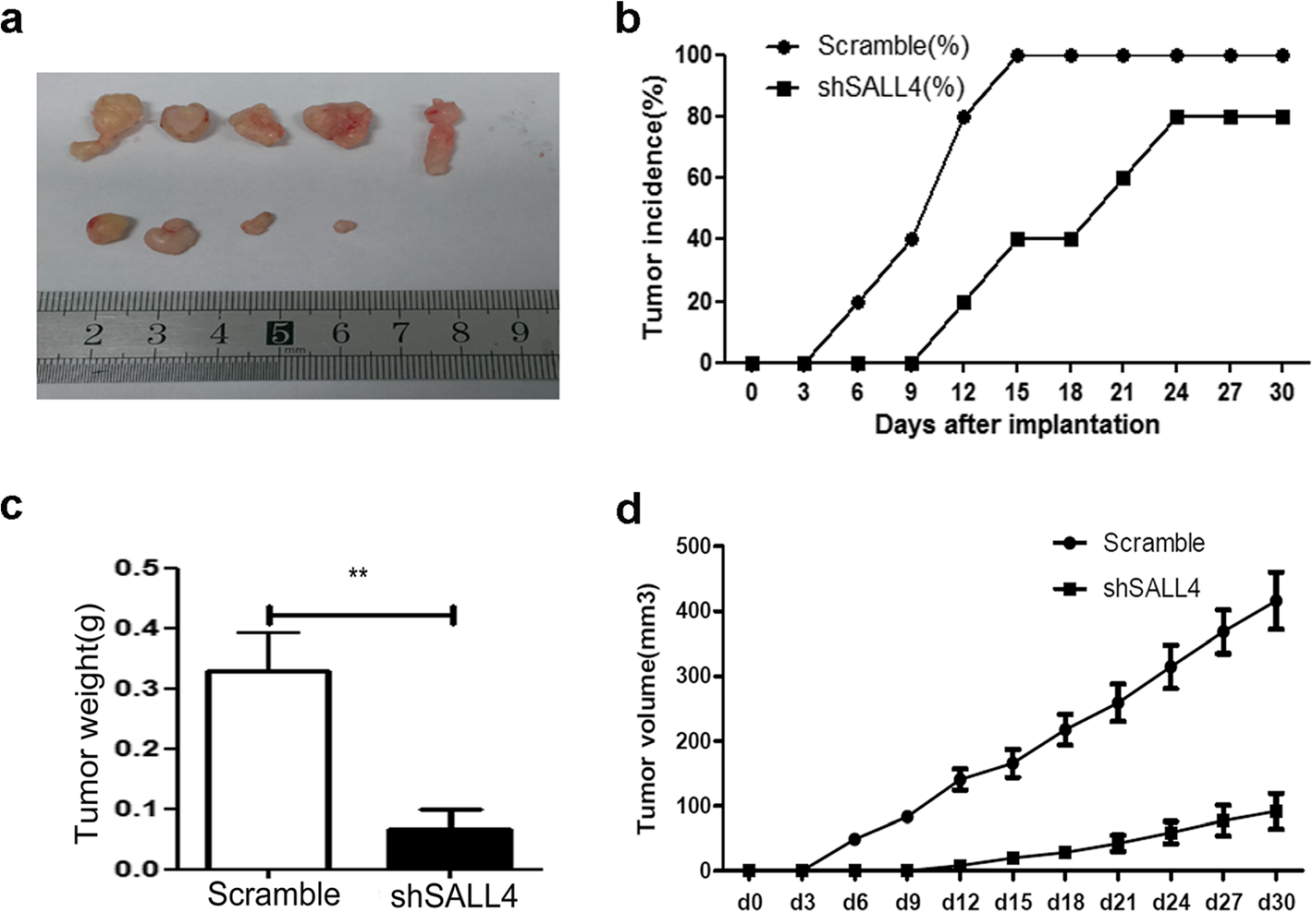
Knock-down of SALL4 inhibits tumor formation in xenograft models in vivo. **a** Tumor volumes in mice injected with shSALL4 TE7 cells were remarkably smaller than the scramble group. Both tumor incidence (**b**) and tumor weight (**c**) were decreased in mice transplanted with shSALL4 TE7 cells compared with those injected with scramble cells. Tumor growth (**d**) was inhibited in mice transplanted with shSALL4 TE7 cells. (**P < 0.01)

To further investigate the influence of down-regulation SALL4 on tumor growth in vivo, we subcutaneously injected the scramble and shSALL4 TE7 cells into nude mice to establish xenograft tumor models. Representative images of tumor size of scramble and shSALL4 groups were shown in Fig. 6a. Scramble cells formed subcutaneous tumors earlier than shSALL4 TE7 cells. In the scramble group, tumor nodule started to form at day 6 after injection, and the tumor incidence reached 100 % at day 15 after injection. However, compared to the scramble group, tumor nodule in shSALL4 group began to form at day 12 and tumor incidence was only 80 % after implantation (Fig. 6b). The tumors grew faster in the scramble group, and the tumor weight was significantly higher than that in the shSALL4 group (Fig. 6c and d). The mean tumor volume in the scramble group reached 430 mm^3^, but only 95 mm^3^ in the shSALL4 group at day 30 after implantation. Taken together, these results suggest that knockdown of SALL4 reduces tumor burden and inhibits tumor formation in vivo.

## Discussion

SALL4 is expressed in fetal organs, including fetal livers, diminished gradually during development of the embryo and silenced in most of the adult tissues but re-expressed in some solid tumors, germ cell tumors and leukemia. Although aberrant activation of SALL4 has been observed in several human malignancies, the potential molecular mechanisms of SALL4 in ESCC remain to be elucidated. Here we reported that SALL4 was significantly up-regulated in above half of the human ESCC tissues, our results were consistent with a recent study on the expression of SALL4 in ESCC [24]. We observed a significant correlation between SALL4 expression and lymph node metastasis, advanced clinical stage and poor survival rate in ESCC, indicating that SALL4 might play a crucial role in ESCC progression. On determining the functional role of SALL4 in ESCC, we provide the first evidence that SALL4 could regulate ESCC cell proliferation, apoptosis, cell cycle, invasion, migration, clonogenicity and stemness. Deng et al. recently found that SALL4 expression was correlated with the Ki67 and CA199 expression in Intrahepatic cholangiocarcinoma (ICC) [25], these two factors are markers for increased proliferation of cancer cells, which may explain why SALL4 influences the proliferation of ESCC cells.

Accumulating evidence suggests that many solid tumors contain a small subset of tumor cells known as CSCs [26], which display stem cell characteristics, including self-renewal and differentiation into a heterogeneous tumor cell population consisting of progenitor cells and more differentiated cancer cells [27, 28]. CSCs are responsible for initiation, progression, recurrence, metastasis and chemo-radiotherapy resistance and therefore considered to be an ideal target for tumor eradication [7, 10]. CSCs have also been identified by their ability to form tumorspheres in serum-free medium containing specific growth factors [29]. However, the reports of CSC markers in ESCC are quite limited. Recent studies demonstrated CD271 and ALDH1 served as CSC markers in ESCC [30, 31]. In this study, we found that SALL4 was related to cancer stemness features, such as colony ability, chemoresistance and EMT phenotype. Here, a strong correlation between thesphere-forming capacity and SALL4 expression level was discovered and the expression of SALL4 was remarkably increased in spheres. Moreover, knockdown of SALL4 abolished sphere formation capacity and decreased expression of CSC markers, such as CD44, CD133 and ALDH1 in TE7 cells. It has been reported that SALL4, combined with Sox2, Oct4 and Nanog, plays a pivotal role in maintaining the stemness of embryonic stem cells (ESC)/pluripotent stem cells. As an up-stream regulator, SALL4 could activate the expression of SOX2, Oct4 and Nanog, meanwhile, these proteins can form a transcriptional feedback network to control the development of ESCs [15, 16, 32]. Our results showed that shRNA-mediated SALL4 inhibition induced the loss of stemness in ESCC cells through decreasing the expression levels of Sox2, Oct4 and Nanog, which was in agreement with previous studies. CSC is considered to be responsible for drug resistance in cancer [8]. Here, we found that knockdown of SALL4 in TE7 cells could increase the sensitivity to cisplatin in a dose-dependent manner. This result suggests that SALL4 could be a potential target for ESCC therapy. Inhibition of SALL4 may efficiently eradicate CSCs in ESCC, which benefit patients with chemorisistance and improve their prognosis. With all findings taken together, we hypothesize SALL4 expression contributes to the stemness in ESCC and SALL4 could serve as a functional CSC marker for ESCC.

EMT plays crucial roles during embryonic development, tumor metastasis and invasion and is one of the major molecular mechanisms through which invasion and metastasis are promoted during the oncogenic process [33, 34]. In the present study, we showed that shRNA-induced SALL4 silencing significantly restricted ESCC cell migration and invasion. Besides, shRNA-induced SALL4 inhibited EMT process by elevating the expression of epithelial marker E-cadherin and reducing the expression of mesenchymal marker Vimentin, suggesting that induction of MET may be, at least partially, responsible for the decreased invasion capacities of ESCC. Furthermore, it is well-known that cancer cells undergoing EMT share the properties of stem cell-like cells [35], which could indicate SALL4 as a CSC marker in ESCC. Wnt/β-catenin signaling pathway plays a critical role in regulating the activity of CSCs in various cancers and contributes to the maintenance of the CSC population [36, 37]. Activation of Wnt/β-catenin signaling pathway has been implicated in stem cell self-renewal, maintenance and differentiation [38, 39]. Ma et al. showed that SALL4 could bind to β-catenin in vitro and synergistically enhanced the Wnt/β-catenin pathway in human acute myeloid leukemia (AML) [17]. Shuai et al. found that Wnt/β-catenin pathway downstream target genes c-Myc and Cyclin D1 were activated and were closely correlated with SALL4 expression in Myelodysplastic syndromes (MDS) patients [40]. Hao et al. observed that SALL4 and β-catenin co-located in the nucleus and cytoplasm and interacted in colorectal cancer (CRC) [41]. However, no further experiments were carried out to illuminate the relationship between SALL4 and Wnt/β-catenin pathway in solid tumors. Our findings have demonstrated that SALL4 triggers the Wnt/β-catenin signaling pathway and shRNA-induced SALL4 inhibition can decrease Wnt3a and β-catenin expression at both mRNA and protein levels, suggesting targeting SALL4 may be a promising new strategy to block Wnt/β-catenin signaling pathway and EMT to prevent tumor metastasis in ESCC. Thus, targeting Wnt/β-catenin pathway potentially represents a promising therapeutic approach for the treatment of ESCC. To the best of our knowledge, this is the first report that SALL4 regulates the expression of Wnt3a, a Wnt protein that activates the canonical Wnt pathway. It has been reported that Wnt3a is responsible for stimulating tumor progression in glioblastoma, prostate cancer and colon cancer [42–44]. Once SALL4 was activated, it may trigger downstream target gene Wnt3a to promote tumor progress and metastasis. Though our study revealed the important role of SALL4 in regulating the expression of Wnt3a, the specific mechanism in this process remains unclear. In future studies, we will investigate whether SALL4 could bind Wnt3a directly or there is a crucial molecule between SALL4 and Wnt3a regulation, and the role of Wnt3a in ESCC.

## Conclusions

In summary, our study shows that aberrantly activated SALL4 may contribute to esophageal tumorigenesis by promoting malignant proliferation and inhibiting cell apoptosis, regulating ESCC cell migration, invasion and cell cycle. Besides, SALL4 expression activates Wnt/β-catenin pathway, uncovering a mechanism underlying EMT in ESCC. However, the specific mechanisms driving aberrant SALL4 expression in ESCC remain to be further elucidated. Taken together, we propose that SALL4 would be a functional CSC marker, a crucial marker for prognosis and an attractive candidate for target therapy of ESCC.

## Abbreviations

CCK8, cell counting kit-8; CSC, cancer stem cell; EMT, epithelial-mesenchymal transition; ESCC, esophageal squamous cell carcinoma; MET, mesenchymal-epithelial transition; SALL4, sal-like protein 4; shRNA, short hairpin RNA.
